# Classroom Standing Desks and Time-Series Variation in Sedentary Behavior and Physical Activity among Primary School Children

**DOI:** 10.3390/ijerph16111892

**Published:** 2019-05-29

**Authors:** Tetsuhiro Kidokoro, Yasuo Shimizu, Kanako Edamoto, Michael Annear

**Affiliations:** 1 Department of Health & Physical, Education College of Arts & Science, International Christian University, 3-10-2 Osawa, Mitaka, Tokyo 181-8585, Japan; syasuo@icu.ac.jp (Y.S.); annear@icu.ac.jp (M.A.); 2 Department of Education, Faculty of Letters, Kanazawa Gakuin University, 10 Sue-machi, Kanazawa, Ishikawa 920-1392, Japan; edamoto@kanazawa-gu.ac.jp

**Keywords:** height-adjustable desks, sitting time, ActiGraph, intervention, hourly variations, school-age children, health

## Abstract

The purpose of the present study was to examine the effects of height-adjustable standing desks on time-series variation in sedentary behavior (SB) among primary school children. Thirty-eight children aged 11–12 years (22 boys and 16 girls) from two classes at a primary school in Nagano, Japan, participated in this study. One class was allocated as the intervention group and provided with individual standing desks for 6 months, and the other was allocated as the control group. Time spent in SB, light-intensity physical activity (LPA), and moderate-to-vigorous-intensity physical activity (MVPA) was measured using accelerometers (ActiGraph) at baseline and follow-up. Time spent in SB was significantly lower by 18.3 min/day on average in the intervention class at follow-up (interaction effects: *F*_(1, 36)_ = 4.95, *p* = 0.035, η^2^ = 0.082). This was accompanied by a significant increase in time spent in MVPA (+19.9 min/day on average). Our time-series analysis showed significant decreases in SB during school time, while no change in SB was found during non-school time. This result indicates that the use of standing desks promotes an overall reduction in SB with no compensatory increase during non-school time.

## 1. Introduction

Sedentary behavior (SB; sitting, lying, reclining, and expending ≤1.5 metabolic equivalents [METs] [1]) is a global problem in modern societies, including Japan [2,3]. A growing body of evidence shows that SB adversely affects cardiometabolic risk markers [4,5]. In addition, it has been reported that high levels of SB are associated with lower self-esteem and academic achievements in children [4,5]. Furthermore, patterns of SB appear to persist from childhood into adolescence and adulthood [6,7], which suggests the need for early life intervention. Effective interventions that aim to reduce SB in childhood are crucial for the current and future health of young people, and schools may provide a helpful setting.

Japanese primary school-aged children spend ≥50% of their waking time in classrooms [8], where they are expected to sit throughout most lessons. Consequently, the classroom is conducive to high levels of SB. Previous studies have shown that children spend 50–70% of their class time in SB and sit for longer periods of time during school hours compared to non-school hours [9,10,11]. Changing the classroom environment into a more active one, therefore, can be a potential strategy to reduce overall SB. One possible way to reduce classroom SB is by replacing standard school desks (i.e., seated desks) with height-adjustable desks (i.e., standing desks), which allow children to study while they are standing and moving. This strategy potentially reduces SB by displacing it with light-intensity physical activity (LPA) (e.g., standing or slow walking) and/or moderate-intensity physical activity (MPA) (e.g., walking). Preliminary evidence shows that the introduction of standing desks decreases SB time among primary school children [11,12,13,14]. Additionally, it has been reported that the introduction of standing desks has beneficial effects on cognitive function and academic achievements [15,16,17] as well as reducing the risk of overweight and obesity by increasing energy expenditure [18,19].

In spite of many reports of the positive effects of standing desks on SB [11,12,13,14], the potential effects on time-series variations in SB have not been examined. Specifically, previous studies predominantly used total daily SB as a main outcome [11,12,13,14]; however, no studies have examined the potential effects of standing desks on hourly variations in SB. Understanding how and when the effects of the standing desk interventions at school occur is essential for evaluating their effectiveness and for the potential design of future standing desk interventions. Therefore, the purpose of the present study was to examine whether the introduction of standing desks can decrease SB and increase physical activity (PA) in school children. Additionally, the study aimed to assess whether changes in school-time activities affect non-schooltime activities. Given the positive effects reported from previous studies [11,12,13,14], we hypothesized that introducing standing desks during school time would increase total PA level.

## 2. Materials and Methods

### 2.1. Participants

Participants were recruited from a public primary school in Saku-city, Nagano, Japan. The school provided two classroom cohorts for the study. In consultation with the head and assistant head teachers, Year 6 children (11–12-year olds) were selected for participation. The parents/guardians of the participants received a letter explaining that the school was replacing existing desks with height-adjustable standing desks in an intervention class. The parents/guardians provided written, informed consent for their children to participate in the study. The study was conducted in accordance with the Declaration of Helsinki and approved by the International Christian University advisory committee (project identification code: 2018-16).

### 2.2. Study Design

The intervention was conducted over a six-month period from the middle of July to the end of December 2018. The present study was a quasi-experimental study with one class being allocated as the intervention classroom, and the other being allocated as the control classroom. In the intervention classroom, comprised of 22 children (13 boys and 9 girls), participants were provided individual, height-adjustable standing desks (Stafit, Okamura Co., Japan) during the intervention period. The standing desks were specifically developed for school-aged children who could adjust the desk height to suit their stature. Additionally, the desk also had wheels, and children could easily move the desks without lifting them. Standard classroom chairs remained at the desks throughout the study, so seated work was also an option. Before the introduction of the desks, the teacher of the intervention class received two face-to-face training sessions on desk use to explain the operation and potential benefits of the standing desks. Session one provided evidence-based information concerning the diverse health and educational benefits of standing desk use. The second session focused on how to use a standing desk within a classroom setting. Furthermore, all teachers received a manual developed by the research team based on previous studies [20], which explained the potential benefits and provided practical examples of using the standing desks (e.g., group work, presentations, and discussions). The participants in the intervention classroom also received an educational lesson explaining health and potential academic benefits of using standing desks to help them to understand and adjust to the change. Although the research team recommended that the participants and teachers avoid prolonged sitting times, the duration and frequency of standing were decided by the participants according to their preference and lesson characteristics. No other environmental and curricular changes were made in the intervention class. The control class, with 21 children (14 boys and 7 girls), was requested to continue with their usual practice of using traditional seated and non-movable desks with no environmental changes. In Japanese primary schools, the academic curriculum is standardized according to government guidelines [21]. In this study, the academic curricula in both classes was identical and no curriculum change was made during the intervention period in either class.

### 2.3. Measurements

Measurements were taken in both classrooms at baseline (prior to desk installation; June 2018) and 19–20 weeks after desk installation (December 2018). The follow-up period was decided after consideration of the school schedule (avoiding school events, i.e., sport and culture festivals). SB and PA were measured by three-axis accelerometers (ActiGraph wGT3X-BT, LLC., Pensacola, FL, USA). The accelerometers have been shown to be valid and reliable activity monitors for measuring SB and PA in children [22,23]. The participants were asked to wear the accelerometer on the right side of their hip using a belt for five consecutive school days (Monday to Friday) except when sleeping or during water-based activities (e.g., showering or swimming). Data were collected in 15 s epochs. Non-wear time was defined as a period of ≥60 min of continuous zero counts as recorded on the ActiGraph [24]. Only the participants with ≥10 h of wear time per day for a minimum of four days were included in the analyses [25]. Evenson’s cut-off points [22] were used to categorize the activities into three levels: SB, <101 counts per minute (CPM); LPA, 101–2295 CPM; moderate-to-vigorous-intensity PA (MVPA), >2295 CPM. The collected data were analyzed using the ActiLife software, version 6.13.3 (ActiGraph, LLC., Pensacola, FL, USA).

### 2.4. Questionnaire Data

At the follow-up, the participants were asked to complete a questionnaire that asked about their perceptions and experiences of the standing desks. Additionally, the participants were asked to respond whether they liked classes with the standing desks and whether they would like to continue using the standing desks. The participants responded to these items using a four-point Likert-type scale ranging from strongly agree to strongly disagree. These items and responses are presented in Table 1.

The participants were also asked to self-report the extent to which they used the standing desks. Here, the participants were asked to respond to the following question: “On average, how often do you use the standing desk?”, possible answers included, “Never (coded as 0)”, “Rarely (coded as 0.5)”, “Once a week (coded as 1)”, Twice a week (coded as 2)”, “three times a week (coded as 3)”, “four times a week (coded as 4)”, or “Every day (coded as 5)”. The participants were also asked to respond to the following question: “When you use the standing desk, how long do you use it for during a class?” The students answered from “0 to 5 min (coded as 2.5)”, “5 to 15 min (coded as 10)”, “15 to 30 min (coded as 22.5)”, or “30 to 45 min (coded as 37.5)”. Following the collection of these metrics, we calculated the average standing desk use time per day using the formula below:

L = (*f* × *l*)/5 (1)

where *L* is the average standing desk use time per day (min/day), *f* is the frequency (0–5 times/week), and *l* is the length (2.5–37.5 min/day). Moreover, the participants were asked to respond to two further questions: “In which subject do you stand up the most? (multiple choices)” and “When do you stand up the most? (multiple choices)”. A summary of all responses and the average standing desk use time per day is presented in Table 2.

### 2.5. Data Analysis

To examine any differences between the intervention class and control class, independent t-tests were performed. A chi-squared test was used to examine differences in the proportion of participants taking part in sport club activities at baseline in the two classes. The proportions of accelerometer wear time in which the participant displayed SB, LPA, and MVPA were calculated for each student separately to account for differences in wear time. Two-way analysis of variance (ANOVA, group × time) was used to examine the differences in changes in activities at baseline and follow-up in the two classes. Effect size (η^2^) in ANOVA was calculated using the formula below:

η^2^ = (SS effect)/(SS total) (2)

where SS effect is the sum of squares for each of the main effects and the interaction, and SS total is the total sum of squares for all effects, interactions, and errors in ANOVA.

Hourly activities were ascertained by calculating the average percentage of activity time per hour. For example, activity at 7:00 a.m. was ascertained by calculating the average percentage of the time spent in activities between 6:30 a.m. and 7:30 a.m. The participants were at school from 8:20 a.m. to 4:00 p.m. (normal Japanese public-school hours), which included three breaks (10:40 a.m. to 11:00 a.m., 12:55 p.m. to 1:40 p.m., and 1:45 p.m. to 2:00 p.m.). Two-way ANOVA (PRE-POST × time) was used to examine the differences in hourly activities in the two classes. On the basis of the effect size (Cohen’s d) of 0.66 reported from a previous study [12], ≥13 participants in each group were required to detect the potential effects of introducing standing desks with 80% power at an alpha level of 5% [26]. Statistical analyses were conducted using SPSS version 24 (SPSS, Inc., IBM, Armonk, NY, USA). Differences were considered statistically significant at *p* < 0.05 and where appropriate effect sizes were reported.

## 3. Results

Out of 22 students in the intervention class, 4 (18.2%; 4 boys) did not provide valid ActiGraph data at baseline and follow-up. Out of 21 students in the control class, 1 (4.8%; 1 boy) did not provide valid ActiGraph data at baseline and follow-up. There were no significant differences in the percentage of the validated data received from the both classes (i.e., participation rates were equivalent, chi-squared test, *p* = 0.170). The final samples, therefore, included 18 students (9 boys and 9 girls) in the intervention class and 20 students (13 boys and 7 girls) in the control class.

There were no differences between the participants in the two groups with regard to MVPA, step counts, accelerometer wear time, and the proportion of participants taking part in sport club activities at baseline (Table 3). However, the proportion of time spent in SB in the intervention class was significantly lower than that in the control class (*p* = 0.023). The participants in the intervention class spent significantly more time in LPA than those in the control class (*p* = 0.005).

**Figure 1:**
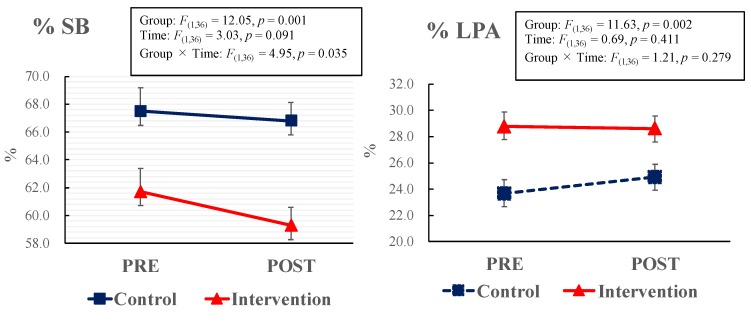

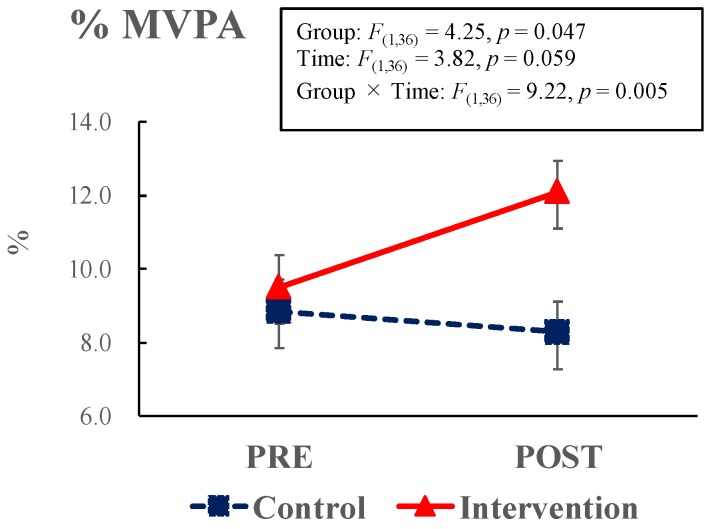
Changes in physical activity and sedentary behavior between intervention and control classes. Data are presented as percentages and standard error. Two-way ANOVA (group × time) was performed to examine the changes in activity levels between baseline and follow-up in the two classes. If the interaction was significant, post-hoc analysis was performed to examine the simple main effects in each factor.

Changes in activity between baseline and follow-up in the both classes were analyzed (Figure 1). SB in the intervention class was significantly decreased at the follow-up, while no change was found in the control class (*F*_(1, 36)_ = 4.95, *p* = 0.035, η^2^ = 0.082). SB decreased by 18.3 min/day between baseline and follow-up in the intervention class. Additionally, MVPA in the intervention class was significantly increased at the follow-up, while no change was found in the control class (*F*_(1, 36)_ = 9.22, *p* = 0.005, η^2^ = 0.213). The MVPA increased by 19.9 min/day in the intervention class. No significant changes in LPA were found for both classes. (*p* = 0.279).

**Figure 2:**
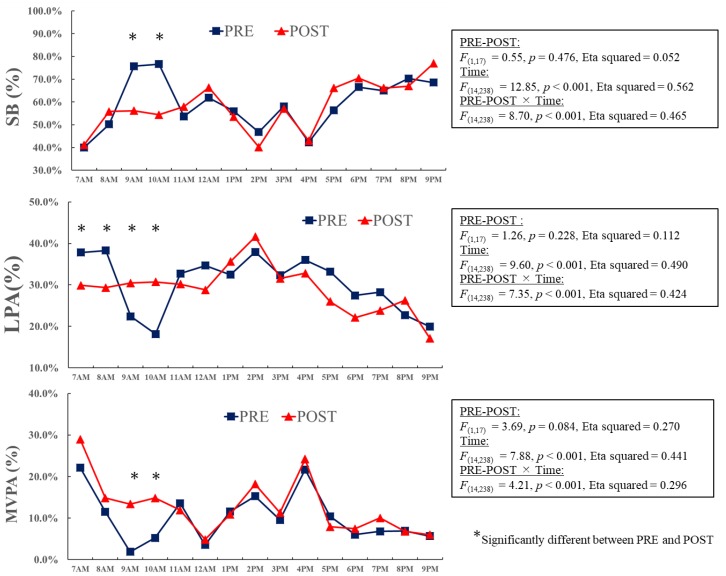
Hourly variations in activity levels in the intervention class at baseline and follow-up. Data are presented as percentages. Two-way ANOVA (PRE-POST × time) was performed to examine the hourly variations in activity levels in the intervention class at the baseline and follow-up. If the interaction was significant, post-hoc analysis was performed to examine the simple main effects in each hour.

**Figure 3:**
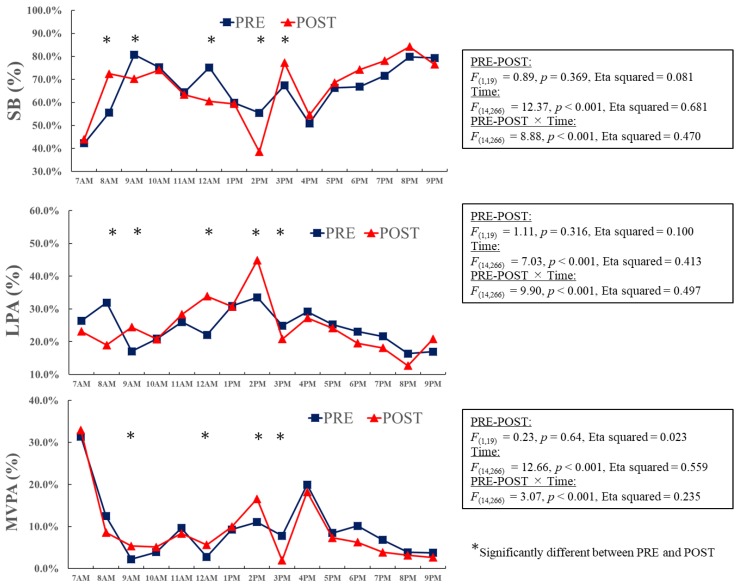
Hourly variations in activity levels in the control class at baseline and follow-up. Data are presented as percentages. Two-way ANOVA (PRE-POST × time) was performed to examine the hourly variations in activity levels in the control class at the baseline and follow-up. If the interaction was significant, post-hoc analysis was performed to examine the simple main effects in each hour.

In order to understand the changes in activity in the intervention class, hourly variations were analyzed at baseline and follow-up (Figure 2). There were significant differences in SB, LPA, and MVPA (all *p* < 0.05) between the baseline and follow-up measurements. In particular, SB at 9 a.m. and 10 a.m. was significantly lower at follow-up among the intervention group. In contrast, MVPA and LPA during the same period were significantly higher at follow-up (all *p* < 0.05). There were no significant differences in activities at other times (Figure 2). Hourly variations in activities were also analyzed in the control class at baseline and follow-up (Figure 3). There were significant differences in SB, LPA, and MVPA (*p* < 0.05) between the baseline and follow-up measurements at 8 a.m., 9 a.m., 12 p.m., 2 p.m., and 3 p.m.. However, no clear pattern of change in the activity levels was found in the control class (Figure 3).

The decrease in SB among the participants in the intervention class was accompanied by positive attitudes toward the standing desks (Table 1). The questionnaire results showed that the 66.7% of children reported enjoying classes using standing desks, and 72.2% of children expressed willingness to continue using their standing desks. Moreover, 66.7% of children felt that they could express their thoughts more effectively, 77.7% of children found it easier to work, and 97.8% of children felt less sleepy when using standing desks. In contrast, only 11.2% of children felt fatigued in the standing classroom.

Standing desk usage in the intervention class was also assessed using a questionnaire (Table 2). The average self-reported standing desk usage time was 21.4 ± 5.9 min/day (these data compare favorably to ActiGraph values for recorded increases in MVPA). Furthermore, the participants reported changing their posture 1.8 ± 0.8 times during a class. The most popular subject for using the standing desks was Japanese language class (94.4%), followed by social studies (77.8%), ethics (66.7%), art and handcrafts (50.0%), calligraphy (44.4%), art (22.2%), and mathematics (5.6%). Additionally, the questionnaire results showed that 94.4% of children used the standing desks during group activities, 44.4% mentioned using the standing desk when making handcrafts, and 33% mentioned that they used the standing desks when talking with classmates.

## 4. Discussion

This paper is the first to report the effects of standing desks in a classroom setting by focusing on time-series variations in SB among primary school children. The present study showed that standing desks significantly decreased SB and increased MVPA. Additionally, our time-series analysis showed that standing desks significantly decreased SB during school time, with no compensatory change in SB during non-school time. This resulted in overall, significant reductions in total SB.

The extent of the SB reductions after the introduction of the standing desk was substantial (−18.3 min/day) and accounted for by an equivalent increase in MVPA (+19.9 min/day) in the intervention class. The reduction in SB found in our study was smaller compared to SB reductions reported by previous studies using standing desks in a similar setting (−55 to −26 min/day) [11,12,13,14]. PA guidelines recommend that children spend a minimum of 60 min each day in MVPA [27], suggesting that the 19.9 min/day increase in MVPA is meaningful. Indeed, our work suggests that standing desks can facilitate an accrual of 33% of daily recommended activity for Japanese children. Although it is difficult to estimate the clinical importance of decreased SB from the present study, a study in adults showed that reallocating just 30 min/day of SB to MVPA was associated with up to a 10.7% improvement in cardio-metabolic biomarkers [28].

Our findings are consistent with the findings from previous studies, which demonstrated that the introduction of standing desks decreased the total time spent in SB among primary school children [11,12,13,14]. The novel finding of this research is that no compensatory change in SB occurred during non-school time as a result of school time SB reduction. This suggests that students are not becoming unnecessarily fatigued by using standing desks and that standing desks can be easily integrated into educational settings. It has been reported that school-based PA interventions, such as the increased number of mandatory physical education program, often do not transfer into overall increases in PA levels due to an associated negative effect on non-school time PA [29,30,31]. The compensatory changes in SB reported in other studies might be explained by children in PA intervention reducing their commitments to PA opportunities outside of school if they perceive that they have been sufficiently active at school [29]. In that sense, compared to PA interventions, reducing SB using standing desks might have been less demanding (or less perceptible as an activity intervention) for the children in our study. As a consequence, they may not have noticed an increase in their activity during school hours. Supporting this contention, our questionnaire survey revealed that only 11.2% of children felt fatigued after using the standing desks, suggesting that the intervention was not physically demanding for most participants. This may also be due to the adjustable nature of the desk and the freedom to sit or stand as the students desired. This is in line with a previous study, which reported similar results regarding the reported physical demands of standing desks [32] and with a literature review showing small but significant positive effects of interventions designed to counter SB on total time spent in SB by children and adolescents [33]. Furthermore, a systematic review examining the effectiveness of SB intervention strategies concluded that installing standing desks in classrooms is a promising strategy among numerous other SB intervention strategies [34]. This is likely because the school classroom offers plenty of room for improvement as an activity intervention setting due to the long periods of seated time in class compared with non-school hours [9,10,11].

The present study demonstrated that SB reduction could be accounted for by an increase in MVPA in the intervention class. This finding is inconsistent with a previous meta-analysis, which argued that there is no association between SB and MVPA in young people, and these behaviors do not directly displace one another [35]. Accounting for the energy cost of human PA, walking can be categorized as MPA, while standing can be categorized as LPA [36]. Given that no significant change in LPA during the intervention was found, introduction of standing desks (movable type) might encourage participants to move (i.e., walk or move their desks) around rather than encouraging them to use the desks in solely a fixed, standing position. Supporting this contention, the results of our follow-up survey showed that group work activity was the most popular activity with the standing desks as 94.4% of children mentioned that they used the standing desks in this context. Only a few children (≤22.2%) mentioned that they used the desks in a fixed, standing position for activities such as writing and reading. Based on interviews with the classroom teacher, it was apparent that she utilized the standing desks to facilitate interaction among children who could move around with their desks to talk with other classmates especially during group work. Therefore, we suspect that this behavior in particular helped to change SB to MPA, although the potential mechanism should be addressed in future studies.

The present study has a number of strengths. It is the first study to conduct a time-series analysis of the effects of the standing desks among primary school children, which provides better understanding of children’s activity patterns. Our findings might, therefore, be useful for future, domain-specific SB interventions and program evaluations (i.e., within or outside of school settings). Secondly, most studies in this area have been conducted in western counties, including the USA [15,16,18,19], New Zealand [12,13], Australia [11,32,37], and Europe [11,14,17,20], which have different educational systems and socio-cultural expectations for childhood behavior. To the best of our knowledge, ours is the first study to provide data in the context of Asian children. As educational and social backgrounds vary across countries and cultures and intervention methods cannot always be applied to all cohorts, it is important to carry out studies in various populations to provide a comparative perspective.

Despite these strengths, there are several limitations to be considered. First, only one school was included in this study, which limits the generalizability of the results. The next step for this project will be to develop a multi-site randomized controlled trial to address this limitation, which also considers the influence of other variables in the educational setting. For example, the school administrators and the teacher from the intervention class were extremely positive in their sentiments about the introduction of the standing desks into the classroom, and we suspect that our results can, in part, be attributed to the facilitation and support of participating educators [38]. In particular, children’s perceptions of the standing desks might be influenced by the positive sentiments expressed by teachers. Therefore, teacher influence should be considered in future intervention studies, as it may prove to be a powerful mediating or moderating variable when considering standing desk effects on classroom SB. Second, we did not measure all potential confounding variables, such as weight gain, during the intervention period. Increases in body weight, particularly body fat, are known to be negatively associated with PA levels [39]. Therefore, these variables should be considered in a future intervention study.

## 5. Conclusions

The present study showed that the introduction of standing desks in a Japanese primary school classroom reduced SB time and increased MVPA during the school day. In particular, SB during school time was significantly decreased, and no compensatory increases in SB occurred during non-school time in the intervention class. The evaluation of total SB and hourly SB variations enables us to comprehensively understand how school-based activity interventions influence children’s behavior in and out of school.

## Figures and Tables

**Table 1:** Children’s perceptions of the standing desks.

Children’s Perceptions	Strongly Agree	Agree	Disagree	Strongly Disagree
Attitude to the standing desks				
I like using the standing desk during class (% (*n*))	16.7 (3)	50.0 (9)	27.8 (5)	5.6 (1)
I want to continue using the standing desk (% (*n*))	27.8 (5)	44.4 (8)	16.7 (3)	11.1 (2)
In the standing desk classroom…				
I can express my thoughts more effectively (% (*n*))	5.6 (1)	61.1 (11)	22.2 (4)	11.1 (2)
It is easier to work (% (*n*))	44.4 (8)	33.3 (6)	11.1 (2)	11.1 (2)
I am fatigued (% (*n*))	5.6 (1)	5.6 (1)	38.9 (7)	50.0 (9)
I feel less sleepy (% (*n*))	55.6 (10)	38.9 (7)	5.6 (1)	0 (0)

**Table 2:** Standing desk usage in the intervention class.

Standing Desk Usage	Mean ± SD or (% (*n*))
Frequency	
Average standing desk use time (min/day)	21.4 ± 5.9
Average frequency of posture change during a class (time/class)	1.8 ± 0.8
In which subject do you stand up the most? (Multiple choices)	
Mathematics (% (*n*))	5.6 (1)
Social studies (% (*n*))	77.8 (14)
Japanese language (% (*n*))	94.4 (17)
Science (% (*n*))	0 (0)
English (% (*n*))	0 (0)
Music (% (*n*))	0 (0)
Home economics (% (*n*))	0 (0)
Technical course (% (*n*))	0 (0)
Ethics (% (*n*))	66.7 (12)
Calligraphy (% (*n*))	44.4 (8)
Art and Handcraft (% (*n*))	50.0 (9)
When do you stand up the most? (Multiple choices)	
During morning activities (% (*n*))	0 (0)
When I feel sleepy (% (*n*))	22.2 (4)
When I feel tired (% (*n*))	11.1 (2)
When I want to concentrate (% (*n*))	16.7 (3)
During group activities (% (*n*))	94.4 (17)
When I read a book	22.2 (4)
When I write	22.2 (4)
When I make handicrafts	44.4 (8)
When I draw a picture	0 (0)
When I perform calculations (% (*n*))	0 (0)
When I talk with a friend (% (*n*))	33.3 (6)
When I present my ideas to others (% (*n*))	16.7 (3)
When I talk to people at a distance (% (*n*))	33.3 (6)
Other (% (n))	0 (0)

**Table 3:** Characteristic of the participants at baseline.

Characteristic of the Participants	Intervention Class (*n* = 18)	Control Class (*n* = 20)	*p* Value
Age (year)	11.3 ± 0.5	11.3 ± 0.5	0.805
Height (cm)	144.6 ± 7.0	145.3 ± 6.6	0.748
Weight (kg)	38.7 ± 9.7	37.0± 8.5	0.534
BMI (kg/m^2^)	18.3 ± 3.1	17.4 ± 3.3	0.379
%Boys (% (*n*))	50 (9)	65 (13)	0.350
SB (%)	61.7 ± 7.9	67.1 ± 6.1	0.023
LPA (%)	28.8 ± 4.9	24.2 ± 4.4	0.005
MPA (%)	6.2 ± 2.6	5.6 ± 2.2	0.429
VPA (%)	3.3 ± 1.8	3.1 ± 1.7	0.695
MVPA (%)	9.5 ± 3.8	8.7 ± 3.7	0.490
Step count (steps/day)	11,295 ± 3,229	11,796 ± 2,140	0.573
Accelerometer wear time (min/day)	764.5 ± 95.4	827.2 ± 89.6	0.045
Sport club activity (% (*n*))	38.9 (7)	40.0 (8)	0.944

Date are presented as mean ± SD; BMI: body mass index; SB: sedentary behavior; LPA: light-intensity physical activity; MPA: moderate-intensity physical activity; VPA: vigorous-intensity physical activity; MVPA: moderate-to-vigorous-intensity physical activity.
